# Crinkle-Cut Coronary Arteries in a Patient With Granulomatosis With Polyangiitis

**DOI:** 10.1016/j.jaccas.2024.102499

**Published:** 2024-09-04

**Authors:** Noor M.A. Alsammarraie, Alycia C. Bellino, Wadie S. David, Misha A. Khan, Bradley L. Hubbard, David A. Sutter, Steven E. Girard

**Affiliations:** a Department of Internal Medicine, Trinity Health Ann Arbor, Ann Arbor, Michigan, USA; b Department of Cardiology, Trinity Health Ann Arbor, Ann Arbor, Michigan, USA

**Keywords:** cardiac magnetic resonance, coronary vasculitis, granulomatosis with polyangiitis

## Abstract

Granulomatosis with polyangiitis (GPA) is a rare type of small to medium vessel necrotizing vasculitis that usually affects vessels of the upper or lower airways and kidneys. Cardiac involvement in GPA is often subclinical and if clinically significant has been rarely reported, even less so as an initial presentation. We describe the case of a 44-year-old man who presented with what appeared to be inferior ST-segment elevation myocardial infarction and was found to have small vessel vasculitis of the coronary arteries with associated myocarditis as a presenting manifestation of GPA, which was ultimately treated with steroids, rituximab, and avacopan.

## Graphical Abstract

**Figure:**
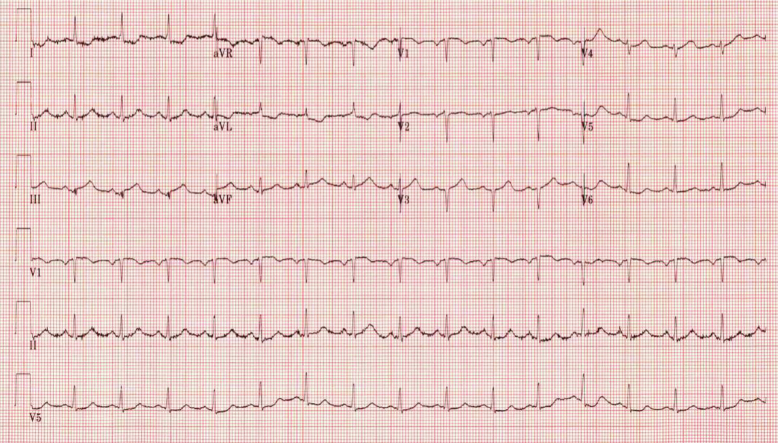

## Background

Granulomatosis with polyangiitis (GPA), formerly called Wegener’s granulomatosis, is an autoimmune inflammatory disorder causing necrotizing vasculitis in small and medium arteries. GPA is the most common antineutrophil cytoplasmic antibody (ANCA)–associated vasculitis, with an annual incidence estimated at 10 to 20 cases per million.^1^ There are 2 types of ANCA: C-ANCA (cytoplasmic antibodies directed against the neutrophil proteinase 3) and P-ANCA (perinuclear antibodies directed against the neutrophils myeloperoxidase). Results of testing for C-ANCA are positive in 88% of patients with active GPA. Typical areas affected in GPA are ears, nose, throat, lungs, kidney, eyes, skin, and peripheral nerves. Cardiac involvement is rare, with only a few cases reported. Most cardiac manifestations are subclinical and remain clinically silent. We present a case of a 44-year-old male patient who presented with chest pain, electrocardiography (ECG) findings of an inferior ST-segment elevation myocardial infarction (STEMI), who ultimately received a diagnosis of GPA, coronary vasculitis, and myocarditis.

**Take-Home Messages**

• Cardiac involvement can be one of the earliest manifestations of GPA, with diffuse coronary small vessel vasculitis resembling myocardial infarction in its presentation.
• CMR is important to detect myocardial involvement in GPA, which can show multiple patterns of LGE (subendocardial, midmyocardial, and epicardial) that are atypical for myocarditis, which usually is characterized by inferior lateral epicardial LGE.

## Case Presentation

**Figure 1:**
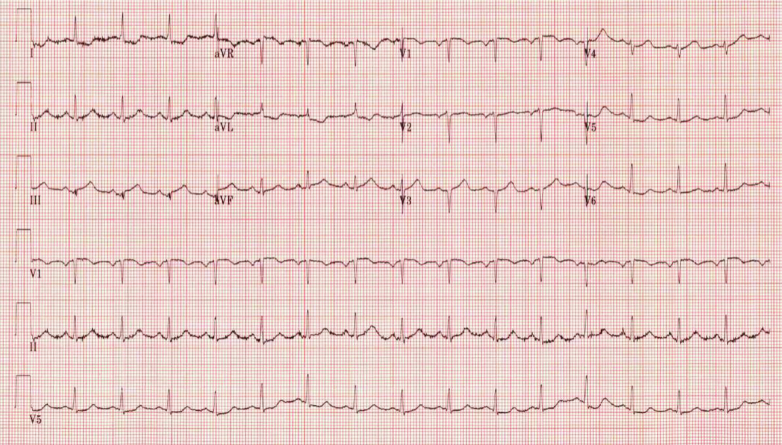
Initial Electrocardiography The tracing shows ST-segment elevation in leads II, III, and aVF, with reciprocal ST-segment depression in lateral leads I, aVL, V_5_, and V_6_.

**Figure 2:**
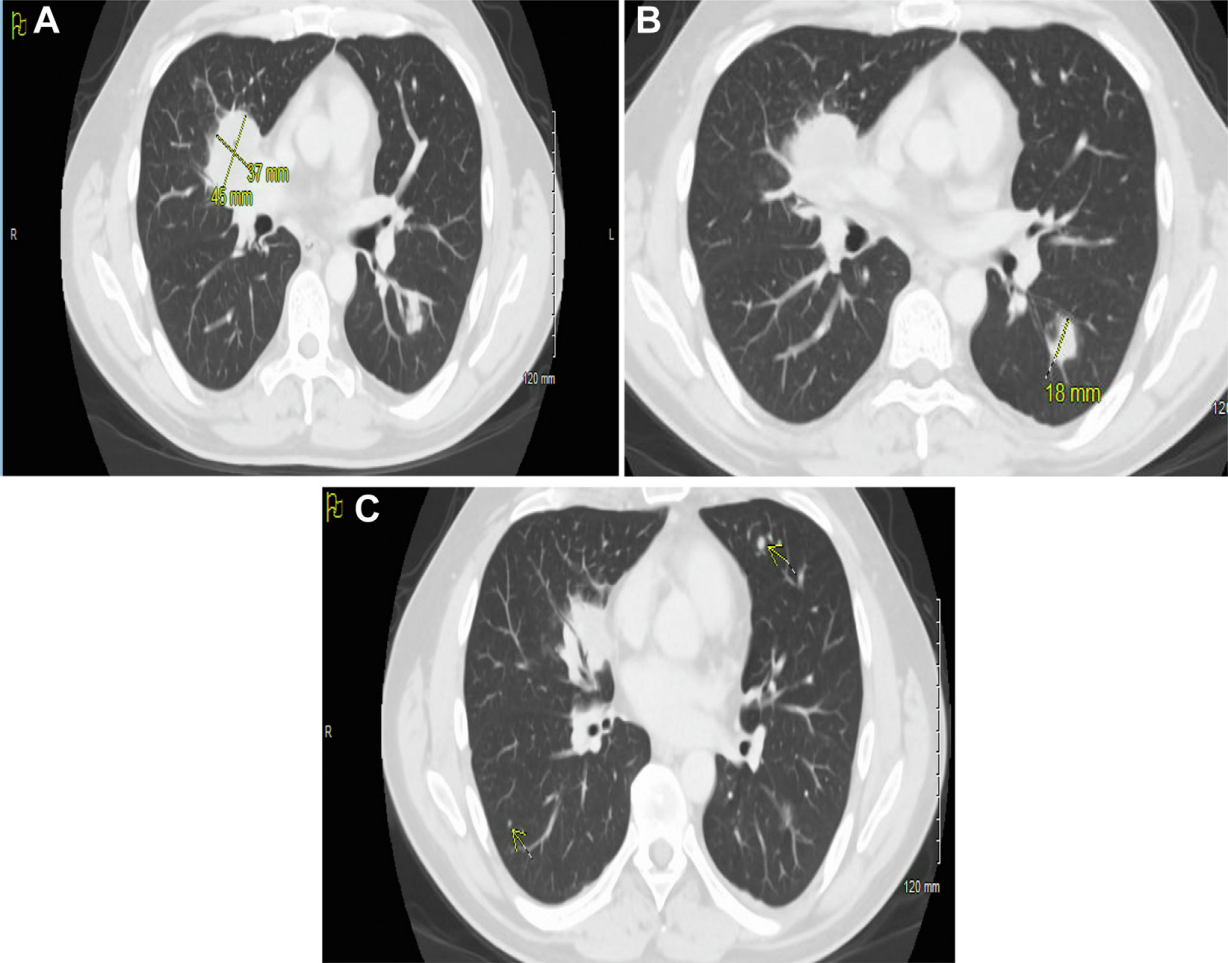
Imaging of Lung Nodules: Computed Tomography Angiography of the Chest (A) An irregular heterogenous mass anteriorly in the right hilum extending into the right upper and middle lobes and measuring 4.5 cm × 3.7 cm. (B) An ill-defined nodule measuring up to 18 mm in the left lower lobe. (C) Arrow on right side of image indicating a tiny 2-mm nodule medially in left lower lobe. Arrow on the left side of image indicating another tiny approximately 2mm rounded nodule in right lower lobe.

**Figure 3:**
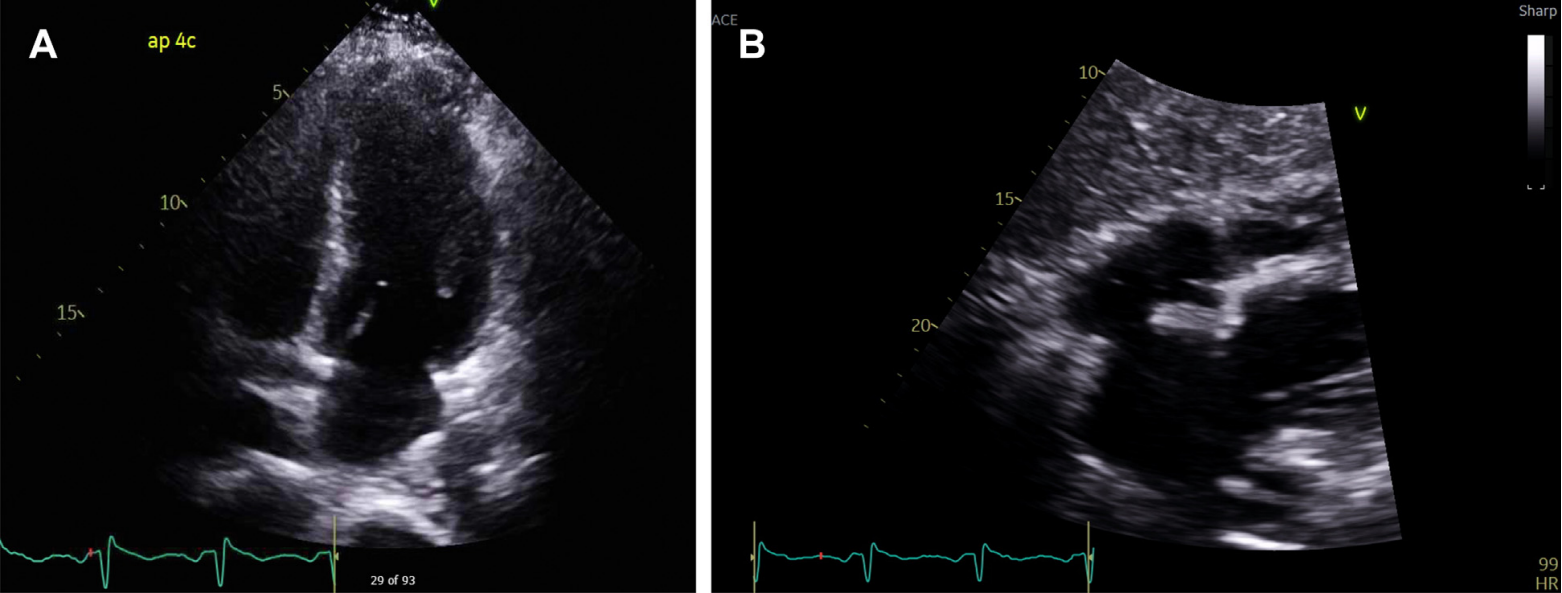
Initial Transthoracic Echocardiogram (A) Left ventricular ejection fraction of 45% with mild left ventricular global hypokinesis. (B) Subxiphoid pericardial view showing a normal left ventricular cavity size and a normal left atrium.

A 44-year-old man presented to the hospital after 2 hours of severe substernal chest pain radiating to the back with associated sweating and nausea. In the days leading to presentation, the patient reported a nonproductive cough and intermittent chest discomfort. Vital signs showed the following: blood pressure, 119/68 mm Hg; heart rate, 90 beats/min; respiratory rate, 20 breaths/min; and oxygen saturation, 94% on room air. Physical examination was notable for severe distress with pale skin and diaphoresis. ECG revealed a 1-mm ST-segment elevation in leads II, III, and aVF, with ST-segment depression in the lateral leads (Figure 1). Initial laboratory tests showed the following: mild leukocytosis, 13.3k/μL; creatinine, 0.81 mg/dL; B-type natriuretic peptide, 647 pg/mL; and high-sensitivity troponin, initially 2,306 ng/L, with an eventual peak of 21,293 ng/L. Given the concern for aortic dissection, urgent chest computed tomography angiography (CTA) was performed. CTA findings were negative for dissection but showed a 5.7-cm lung mass in the right apex, a 5-cm lung mass in the anterior right hilum, and multiple lung nodules (Figures 2A to 2C). Further computed tomography (CT) images of the abdomen/pelvis and head were normal. Transthoracic echocardiography (TTE) revealed a left ventricular ejection fraction of 45% with mild global hypokinesis, rather than the expected regional wall motion abnormality typical of a coronary infarct (Figures 3A and 3B). Because high-quality TTE identified no regional abnormalities typically expected in a type I myocardial infarction, and with nearly complete resolution of symptoms following initiation of medical management, emergent coronary angiography was not performed. This difficult decision was reached after appropriate shared decision making between the patient and the on-call interventional cardiologist. It was thought that avoiding dual antiplatelet therapy would allow for timely lung biopsy for diagnosis, which was urgently needed to initiate appropriate therapy. He was admitted to the cardiac intensive care unit for careful observation and medical therapy.

Our patient’s past medical history was pertinent for tobacco smoking. Review of systems revealed a few months of purulent rhinorrhea, low-grade fever, chills, and night sweats along with a 40-pound weight loss over the past year.

**Figure 4:**
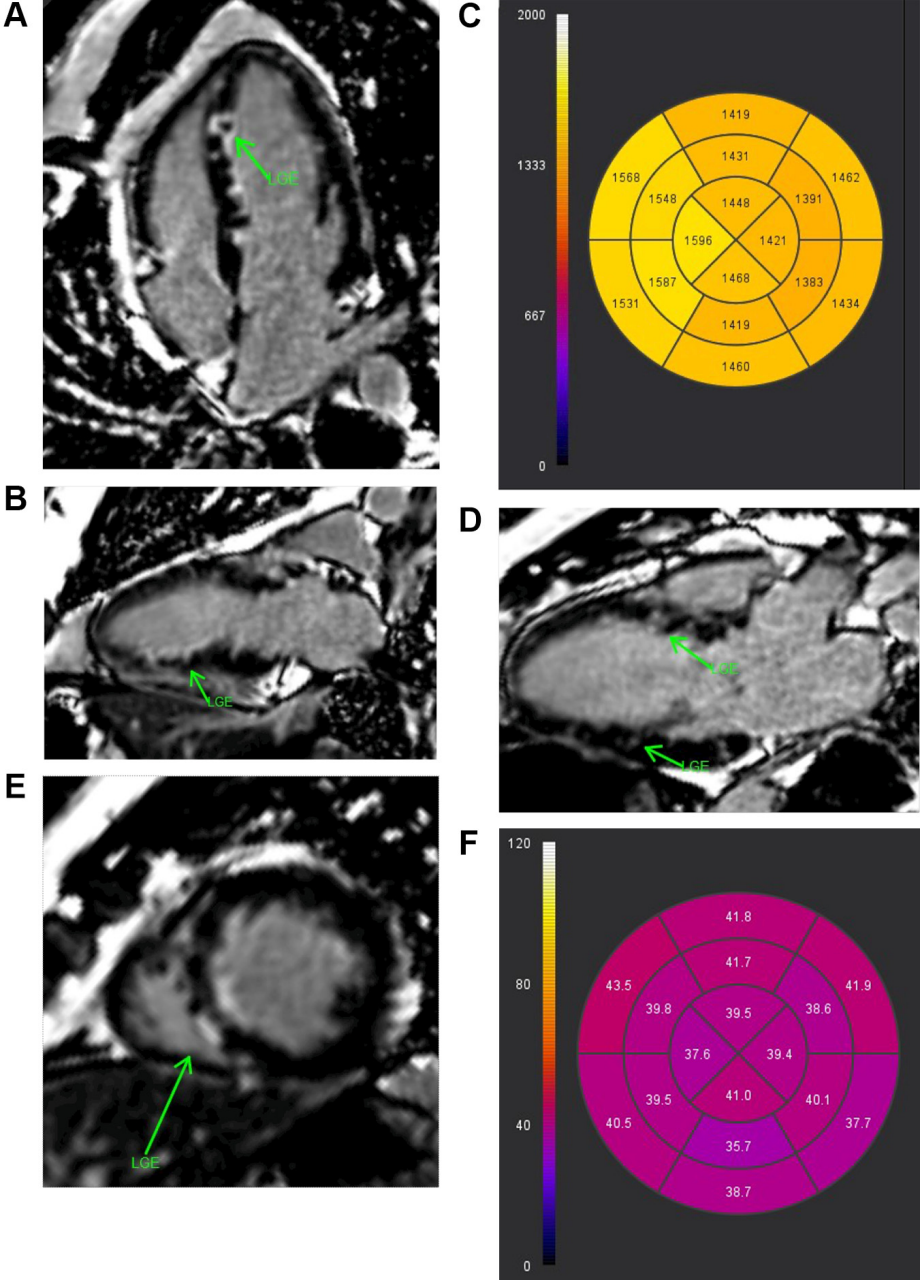
Initial Cardiac Magnetic Resonance (A) A 4-chamber view with arrow highlighting area of late gadolinium enhancement (LGE). (B and C) Multiple 2-chamber views with arrows highlighting areas of LGE. (D) A short-axis view with arrow highlighting area of LGE. (E) T1 mapping. (F) Elevated T2 mapping.

Following 48 hours of intravenous heparin, the patient underwent bronchoscopy with biopsy, which was nondefinitive but negative for malignancy, the initial concern. CT-guided biopsy of a lung mass was then performed, and findings were compatible with granulomatosis with polyangiitis (GPA). Rheumatology consultants recommended further evaluation with antinuclear antibody (ANA) and antineutrophilic cytoplasmic antibody (ANCA). ANA was positive, with a titer of 1:160 with a speckled pattern, and ANCA was positive, with a titer of 1:320. The patient maintained normal kidney function during hospitalization. For further characterization of potential cardiac involvement, we began evaluation for myocarditis and ischemia. Cardiac magnetic resonance (CMR) showed mildly decreased systolic function with an ejection fraction of 43%, multiple patterns of late gadolinium enhancement (LGE) in subendocardial (basal inferoseptal, midinferoseptal, apical), midmyocardial (basal inferoseptal, midinferoseptal, apical), and epicardial (midinferoseptal) distributions, with elevated T2 tissue mapping, and extracellular volume (ECV) suggesting myocardial edema (Figure 4).

**Figure 5:**
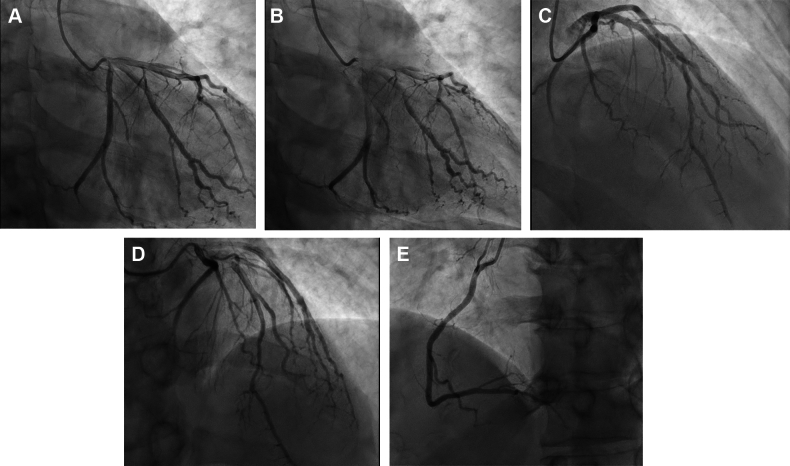
Crinkle-Cut Coronary Arteries Cardiac catheterization imaging showing diffuse small vessel coronary vasculitis. (A and B) Coronary angiogram demonstrating the left coronary artery system. The left anterior descending (LAD) artery exhibits a distinctive “crinkle-cut” appearance characterized by irregular, undulating contour suggestive of segmental stenosis and dilation along the vessel wall that are especially prominent distally. The left circumflex (LCx) artery here is visible in the proximal portion and appears to have less involvement in this view. (C) The distal LAD artery and its smaller branches. This image highlights the impact of vasculitis on the microvasculature, where the damage appears more pronounced. (D) The proximal and mid segments of the LAD distorted vessel architecture. (E) Coronary angiogram demonstrating the right coronary artery “crinkle cut” appearance especially in the mid to distal segment.

To complete the diagnostic evaluation, the patient underwent coronary angiography, which showed small vessel diffuse disease suggestive of vasculitis (“crinkle-cut” appearance) without significant coronary artery disease (CAD) (Figure 5). There were no signs of obstructive disease or active plaque on coronary angiography, thus further supporting the decision not to perform immediate cardiac catheterization.

**Table:** VISUAL SUMMARY

Admitted to the hospital for concern for inferior STEMI
Underwent bronchoscopy, which was negative for malignancy
Underwent CT guided lung biopsy, showing vasculitis
Underwent Cardiac MRI showing EF 43%, multiple patterns of late gadolinium enhancement in subendocardial (basal inferoseptal, mid inferoseptal, apical), mid myocardial (basal inferoseptal, mid inferoseptal, apical), and epicardial (mid inferoseptal) distributions with elevated T2 tissue mapping, extracellular volume suggesting myocardial edema
Started on methylprednisolone with transition to prednisone for treatment of GPA
Underwent coronary angiography, which was unremarkable for coronary artery disease or active plaque
Discharged from the hospital
Started Rituximab by rheumatology
Completed 30-day event monitor with no significant arrhythmias
Started Avacopan
Repeat cardiac MRI showing improvement of myocarditis

The patient was started on methylprednisolone, 500 mg intravenously once daily for 3 days, and was discharged on prednisone, 60 mg daily, with plans to initiate rituximab on an outpatient basis for treatment of GPA. Standard goal-directed medical therapy for heart failure with spironolactone, losartan, and metoprolol was also initiated before discharge.

## Outcome and Follow-Up

Over the next 6 months, rheumatologists treated the patient with 2 cycles of rituximab infusions while tapering the prednisone, with subsequent initiation of avacopan with discontinuation of prednisone. A 30-day event cardiac monitor did not reveal any significant arrhythmias. Surveillance CMR after 6 months showed a marked improvement in both left and right ventricular function, as well as resolution of the myocardial inflammation with normalization of the T2 mapping. However, LGE was still present at the basal midanteroseptum and inferoseptum.

## Discussion

GPA is a type of small to medium vessel necrotizing vasculitis with an annual incidence estimated at 10 to 20 cases per million persons worldwide.^1^ It mainly affects the pulmonary and renal systems. A cohort study conducted between 2006 and 2013 showed that among 517 patients with a diagnosis of GPA, only 17 had cardiac involvement.^2^ Clinical cardiac manifestations of GPA vary over a wide spectrum, including pericarditis, myocarditis, valvulitis, cardiomyopathy, arrhythmias, third-degree atrioventricular block, and myocardial infarction.

This case illustrates both typical and atypical presentations of GPA. The patient’s nasal and upper respiratory symptoms were typical of GPA because they occur in 70% to 100% of patients.^3^ Constitutional symptoms such as weight loss and imaging findings such as lung masses with nodules are nonspecific but typical of GPA. The presentation with acute chest pain, ECG changes initially concerning for inferior STEMI, and elevated troponin, as described here, is a highly unusual cardiac manifestation of GPA. Most cases are subclinical and remain clinically silent.^4^ Despite no atherosclerotic disease seen on coronary angiography, this presentation appeared consistent with acute myocardial infarction. Although GPA usually spares the coronary arteries, inflammation and endothelial dysfunction from GPA can cause accelerated atherosclerosis and an increased risk of CAD in patients with GPA.^5^ The diffuse coronary small vessel vasculitis seen on the left-sided heart catheterization could not explain the inferior STEMI on ECG or the significant rise in troponin. Thus, the rise in troponin was likely caused by myocarditis.

On the basis of CMR findings, it is unlikely that acute coronary arteritis is what caused this presentation and troponin elevation. Acute coronary arteritis will cause subendocardial late gadolinium uptake, as in this patient, but it would be expected to be in a specific coronary artery distribution, which was not seen. There is the potential that if small perforating vessels were involved, it could cause subendocardial late gadolinium uptake with less of a coronary artery distribution.

Studies indicate that most patients with GPA have early subtle myocardial involvement that can be detected only by CMR.^6^ These studies have shown that patients with GPA have significantly higher myocardial T1 mapping with calculation of ECV fraction, which detects edema and subtle inflammation. Increased ECV and T2 mapping are consistent with acute edema, which can be seen in both myocarditis and acute myocardial infarction. Because cardiac catheterization ruled out acute myocardial infarction and subsequent CMR 6 months later showed decreased ECV and T2 mapping, this suggests a resolving acute process, more closely aligning with myocarditis. LGE and mapping techniques for CMR can detect myocardial inflammation and fibrosis in patients with GPA.^7^^,^^8^ Myocarditis typically manifests as inferior lateral epicardial LGE classically in the inferior lateral walls. In our case, initial CMR showed LGE involving the basal inferior septum, the inferior septum, and apical walls and epicardial late uptake, which would suggest possible myocarditis.

According to 2021 American College of Rheumatology/Vasculitis Foundation guidelines, our patient had severe disease resulting from his cardiac involvement. The main goal of treatment is to induce remission in severe organ-threatening disease. Treatment is started with high-dose glucocorticoids (intravenous pulse methylprednisolone, 500-1000 mg/day for 3-5 days, followed by prednisone, 1 mg/kg/day [up to 80 mg daily]). The mainstay of remission induction therapy is rituximab, with clinical remission often occurring after 6 months of therapy. Once remission is induced, the patient receives rituximab every 4 to 6 months for prevention of relapse.^9^ Avacopan is a recently approved medication used to induce and maintain remission in ANCA-associated vasculitis as a replacement for prednisone. It has been shown to be superior in maintaining remission after 52 weeks.^10^ Our patient experienced improvement of cardiac symptoms with prednisone within the first week and achieved clinical and CMR remission with rituximab. He is currently receiving maintenance treatment with avacopan and tapering prednisone. This case did not require Institutional Review Board approval. Written consent was obtained.

## Funding Support and Author Disclosures

The authors have reported that they have no relationships relevant to the contents of this paper to disclose.
